# Random walk with chaotically driven bias

**DOI:** 10.1038/srep38634

**Published:** 2016-12-08

**Authors:** Song-Ju Kim, Makoto Naruse, Masashi Aono, Hirokazu Hori, Takuma Akimoto

**Affiliations:** 1 WPI Center for MANA, National Institute for Materials Science, Tsukuba, Ibaraki 305–0044, Japan; 2 NSRI, National Institute of Information and Communications Technology, Tokyo 184–8795, Japan; 3 Earth-Life Science Institute, Tokyo Institute of Technology, Tokyo 152–8550 & PRESTO JST, Japan; 4 Graduate School of Medicine and Engineering, University of Yamanashi, Yamanashi 400-8511, Japan; 5 Department of Mechanical Engineering, Keio University, Kohoku-ku, Yokohama 223-8522, Japan

## Abstract

We investigate two types of random walks with a fluctuating probability (bias) in which the random walker jumps to the right. One is a ‘time-quenched framework’ using bias time series such as periodic, quasi-periodic, and chaotic time series (chaotically driven bias). The other is a ‘time-annealed framework’ using the fluctuating bias generated by a stochastic process, which is not quenched in time. We show that the diffusive properties in the time-quenched framework can be characterised by the ensemble average of the time-averaged variance (ETVAR), whereas the ensemble average of the time-averaged mean square displacement (ETMSD) fails to capture the diffusion, even when the total bias is zero. We demonstrate that the ETVAR increases linearly with time, and the diffusion coefficient can be estimated by the time average of the local diffusion coefficient. In the time-annealed framework, we analytically and numerically show normal diffusion and superdiffusion, similar to the Lévy walk. Our findings will lead to new developments in information and communication technologies, such as efficient energy transfer for information propagation and quick solution searching.

What if a gambler uses a unconventional coin in which the ‘bias’ (unequal probabilities of obtaining heads or tails) dynamically fluctuates? It is known that the gambler’s success heavily relies on the physical properties of the coin such as the bias. A random walk is a mathematical model for predicting the statistical consequences of successive random choices from multiple options, including the financial success of a gambler who tosses a coin for a bet. To investigate the statistical properties of random walks with fluctuating biases, one can consider two types. One is a ‘time-quenched’ framework, where the bias as a function of time, i.e. a bias time series, does not change for different realisations of the random walks. The other is the annealed version, i.e. a time-annealed framework, where the bias time series can change for different realisations.

A random walk is a simple model of diffusion. In a homogeneous environment, it is characterised simply by the jump length and the probability *p* that a random walker jumps to the right (or equivalently, the probability of a left jump, i.e. *q* = 1 − *p*). When *p* ≠ *q*, there is a bias in the random walk, which generates drift characterised by *p* − *q*. There are typically two types of disordered environments. One is a random energy landscape described by the random potential depths, the so-called quenched trap model (QTM)^1^. In the QTM, while a random walker jumps to the left or right site with equal probabilities, the waiting time for the jump strongly depends on the position. The other is a random walk with a position-dependent bias, so-called Sinai’s model^2^, where the probability *p* depends on the position of the random walker. In Sinai’s model, the probability *p*_*i*_ that the random walker jumps to the right at position *i* is an independent, identically distributed random variable in [0, 1], and the net bias is zero: 
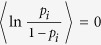
. A combination of the QTM and Sinai’s model is also possible. This model exhibits anomalous diffusion, where the mean square displacement (MSD) increases as 〈*x(t*)^2^〉 ∝ ln^4^(*t*), where *x(t*) is the position of a random walker at time *t*. The models we consider, i.e. the time-quenched and time-annealed frameworks, are different from Sinai’s model as well as the QTM because our models are annealed in the sense that the probability *p* does not depend on the position, while we consider two types of annealing procedures.

Recently, high-frequency trading (HFT) has become dominant in stock markets by algorithmic trading including artificial intelligence^3^,^4^,^5^. This situation would be comparable to the situation in which many trading algorithms for investors (random walkers) stochastically invest according to a time series of the stock price or some stock market index. This perspective of the stock market gives us the time-quenched framework of a random walk, in which investors perform random walks according to a bias time series, which is given equally for all investors. On the other hand, we also consider the time-annealed framework. The physical mechanism of such an annealed fluctuating bias stems from a change in the shape of coin because of collision deformation during a coin toss. This random walk can be represented by the time-annealed framework.

Previously, some of the authors theoretically and numerically demonstrated that chaotic oscillation occurs in a nanoscale system consisting of a pair of quantum dots (QDs), between which energy transfers via optical near-field interactions^6^. The chaotic oscillation occurs at the values of the existence probability of an exciton in one of the QDs when the QDs are connected with an external time delay. It is scientifically and technologically important to elucidate the exact nature of this new oscillatory phenomenon, which we call ‘nanochaos’, since it implies that ultrafast random number generators^6^, problem solvers^7^,^8^, and decision makers^9^,^10^,^11^ can be constructed by the nanoscale elements. These results also inspire us to study the random walk with a temporally fluctuating bias, which is an annealed version of Sinai’s model. Since such a framework has not been studied so far but is relevant to photoexcitation transfer systems, it is physically important to investigate the diffusion.

Here, we consider two types of bias in the annealing procedures. One is a fixed bias, where the bias time series generated by dynamical systems or stochastic processes does not change when taking the ensemble average, i.e. quenched in time. The other is a completely annealed bias, where the bias time series is always different from each realisation when taking the ensemble average. This approach enables us to evaluate how the chaotic dynamics generating the fluctuating bias contribute to diffusion phenomena. In the following, we will present simulation results of random walks using time series in which the fluctuating bias is periodic, quasi-periodic, and chaotic in the time-quenched framework. In the time-annealed framework, we consider a simplified model of a random walk with a fluctuating bias generated by a stochastic process and will present normal and anomalous diffusion analytically. We will provide some discussion of the implications of our results for solution searching.

## Results

### Results in the time-quenched framework

Diffusion is usually characterised by the mean square displacement (MSD). However, when there is a bias (*p* − *q* ≠ 0), the mean displacement and the variance of the displacement are given by 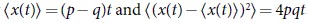. Thus, the diffusivity defined by the degree that the random walker deviates from the mean displacement is characterised by the variance of the displacement. Hence, a time-dependent (instantaneous) diffusivity *D(t*) ≡ 4*p(t)q(t*) fluctuates and crucially depends on the bias time series when we consider a random walk with a fluctuating bias, where *p(t*) is the probability of the right jump at time *t* and *q(t*) = 1 − *p(t*). In the time-quenched framework, the time series of the probability *p(t*) is quenched in time, and we assume that the total bias, i.e. 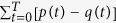, is zero. Because the bias fluctuates and is quenched in time, it is difficult to characterise an effective diffusivity defined by the asymptotic behaviour of the MSD in the long-time limit. However, it is physically natural to expect that the effective diffusion coefficient *D*_*eff*_ is given by the time average of the instantaneous diffusivity: 
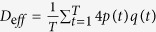
.

To extract the effective diffusion property, we consider the MSD and variance of the displacement based on the ensemble and time averages. If the squared displacement (SD) in a system is ergodic; that is, the time average of SD converges to a constant in the long-time limit, the time-averaged MSD is equivalent to the ensemble average (MSD). However, in some stochastic processes, this equivalence does not hold^12^. Because we do not know the ergodic properties of the SD in our system, we employ the ensemble average of the time-averaged mean square displacement (ETMSD), defined as


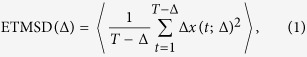


where Δ*x(t*; Δ) ≡ *x(t* + Δ) − *x(t*) is the displacement of the position in [*t, t* + Δ] with the initial condition *x*(0) = 0, 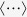 denotes the ensemble average with respect to the noise, and *T* is the total measurement time (*T* = 900,000). Because the total bias, i.e. the sum of *p(t*) − *q(t*), is zero, we did not subtract the bias in the ETMSD. When there is no bias, i.e. *p(t*) = 0.5, the ETMSD exhibits normal diffusion, i.e. ETMSD(Δ) = Δ. The other is the ensemble average of the time-averaged variance (ETVAR) defined as follows:


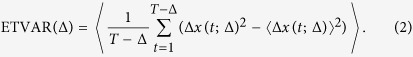


In the ETVAR, a bias in a moving frame [*t, t* + Δ] is actually subtracted.

**Figure 1:**
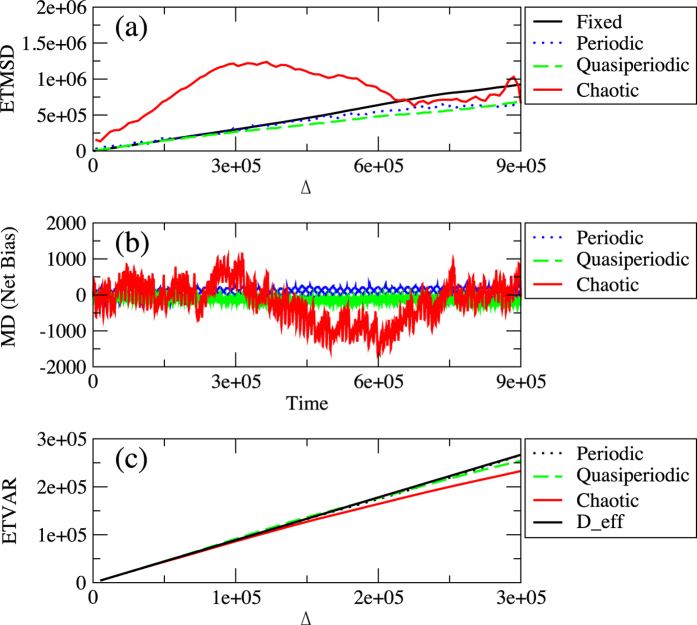
Diffusivity and bias in random walks with a fluctuating bias. (**a**) Ensemble average of the time-averaged mean square displacements, where the bias sequences are generated by fixed (black solid line), periodic (blue dotted line), quasi-periodic (green dashed line), and chaotic (red solid line) time series. (**b**) Average difference between the number of right and left jumps until time *t*, i.e. mean displacement (MD) (‘net bias’). (**c**) Ensemble average of the time-averaged variances, where the bias sequences are generated by periodic (blue dotted line), quasi-periodic (green dashed line), and chaotic (red solid line) time series, and the black solid line represents the theory: ETVAR (Δ) = *D*_*eff*_Δ.

Figure 1(a) shows the ETMSDs, where the bias sequences are shown in Method. The ETMSDs do not monotonically increase, except in the ordinary case (the case with a fixed probability of 0.5). In particular, the ETMSD for the chaotic time series undergoes many large deviations from the linear scaling in a short time interval owing to large fluctuations in the net bias defined by the sum of *p(t*) − *q(t*) (see Fig. 1(b)), although the total bias is zero. Moreover, the overall time dependence of the ETMSDs is proportional to the time, which indicates normal diffusion, as shown in Fig. 1(a). We confirmed that the ETMSD at Δ = 10^6^, 2 × 10^6^, 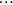 increases linearly with the time when we periodically generate *p(t*) with a period of 10^6^ (not shown). Therefore, the local diffusive nature cannot be characterised by the ETMSD in a short-time interval.

The fluctuations in the net biases are shown in Fig. 1(b). There is a long-time correlation in the time series of *p(t*) for the chaotic one (No. 180); thus, the spectrum contains a high power at low frequencies (not shown). It is noted that the ETMSD of strongly chaotic data such as a logistic map or the No. 180 chaotic data with a destructive time correlation (surrogate data) exhibits similar behaviour to that of no bias, i.e. *p(t*) = 0.5 (monotonic increase in the ETMSD). There exists a huge net bias in a short time interval, although these biases are cancelled in the overall time series owing to the normalisation (see Method).

Because the large fluctuations in the ETMSD originate from the fluctuating bias (Fig. 1(b)), we have to subtract the first moment if we consider the diffusion in the time-quenched framework. Figure 1(c) shows the ETVARs with the bias time series shown in Method. We conclude that the diffusion in the time-quenched framework is normal in general. We also confirm that *D*_e*ff*_ (=0.8999) is very close to the slopes in Fig. 1(c). This implies that the ETVAR successfully characterises the diffusion itself in the time-quenched framework.

### Results in the time-annealed framework

**Figure 2:**
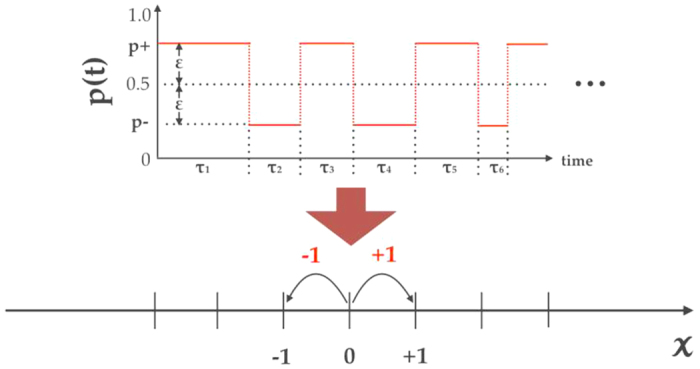
Simplified random-walk model.

Unlike the time-quenched framework, one can assume that the bias at time *t* can be always set to zero in the time-annealed framework because it is possible to assume that the ensemble average of *p(t*) becomes 0.5 for any *t* ≥ 0. Under this assumption, the ETMSD can characterise the diffusive properties, while it is equivalent to the ETVAR in the time-annealed framework. In this section, we consider a simple model related to a random walk with a fluctuating bias, which can generate a variety of diffusion types, as shown in Fig. 2. The model is a simple generalisation of a Lévy walk, in which a random walker performs a two-state biased random walk with a random persistence time. The probabilities of going to the right and the left are given by 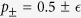 (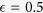 corresponds to the ordinary Lévy walk), where the subscript represents the state, i.e. the + (the right jump) or − state (the left jump). The MSDs can be analytically calculated for several persistence time distributions. Using the analytical results of the model, we can understand that the overall properties in nanochaos data we treat in this paper are all classified as normal diffusion (see Eq. (24)).

#### Simplified random-walk model

In a Lévy walk, a random walker moves to the right or left with a constant velocity for a continuous random persistence time. After the persistence time, the random walker can change the direction. Instead, a random walker in our model performs a biased random walk with the probability 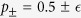 for a continuous random persistence time. In what follows, we consider the continuous-time version of the random walk. Thus, we use the following propagator during the biased random walk with the state ±:


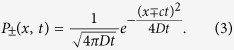


We note that the diffusion coefficient *D* and the velocity *c* are expressed as 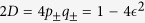 and 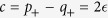, respectively, where *q*_±_ = 1 − *p*_±_. We use *ρ(t*) as the probability density function (PDF) of the persistence times.

#### General framework

Let *Q*_±_ (*x, t*) be the joint PDF of finding a random walker at position *x* at time *t*, and the states changes from 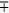 to ± exactly at *t*. Further, let *R*_±_ (*x, t*) be the PDF of finding a random walker with the state ± at position *x* at time *t*. Then, we have the following Montroll-Weiss equations (deterministic change of state)^13^,^14^:


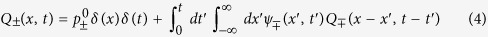


and


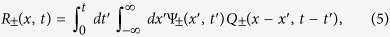


where 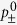 is the probability of the initial state (±), *ψ*_±_(*x, t*) = *P*_±_(*x, t)ρ(t*), and 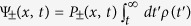. We assume that the initial position is *x* = 0 and that the initial persistence time distribution is the same as *ρ(t*). Note that the initial persistence time distribution is different in general^15^. In particular, when there is an equilibrium distribution for the initial persistence times, the MSD depends clearly on the initial persistence distribution^16^,^17^. Using the Fourier-Laplace transform of *Q*_±_(*x, t*) with respect to *x* and *t* in Eq. (4), we have 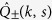. Then, the substitution of this into the Fourier-Laplace transform of 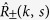 in Eq. (5) gives


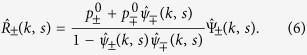


When 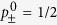, we have


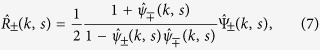


and *R(x, t*) ≡ *R*_+_(*x, t*) + *R*_−_(*x, t*) becomes


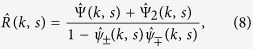


where 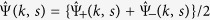, and 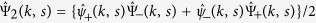. We note that 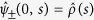, 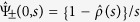, 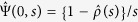, and 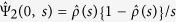.

#### Persistence time distribution (periodic case)

Here, we consider that the persistence time of the state is constant (*τ*_0_): *ρ(t*) = *δ(t* − *τ*_0_). By Eq. (4), we have


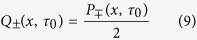


and


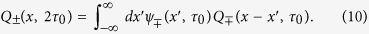


The Fourier transform with respect to *x* is


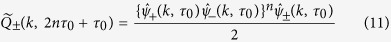


and


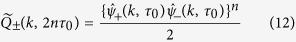


for 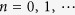.

#### Persistence time distribution (Stochastic case)

Here, we consider three cases for the PDFs of the persistence times whose Laplace transforms are given by

1. *α* = 2: 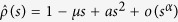,
2. 1 < *α* < 2: 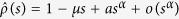,
3. *α* < 1: 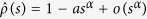,

where *μ* is the mean persistence time and *a* is an arbitrary value. The mean persistence time diverges for case (3), and the second moment of the persistence time diverges for cases (2) and (3). For example, cases (2) and (3) correspond to


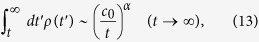


where *c*_0_ is a microscopic scale parameter. Because


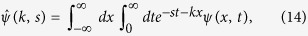


the derivative with respect to *k* gives


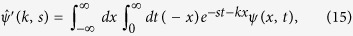


implying 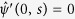. Moreover,


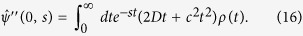


For *α* < 2, one can neglect the first term in the asymptotic limit:


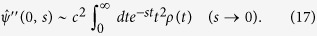


In a similar manner, we have 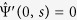 and


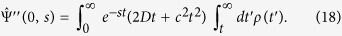


For *α* < 2, one can neglect the first term in the asymptotic limit:


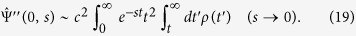


#### Mean square displacement

The Laplace transform of the mean displacement is given by


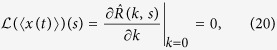


where the symbol 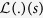 means the Laplace transform with respect to *t*: 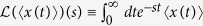. The Laplace transform of the MSD is given by


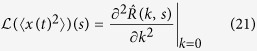



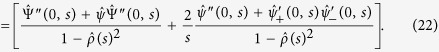


For case (1),


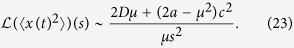


The inverse transform is


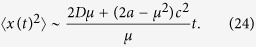


For case (2),


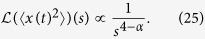


The inverse transform is


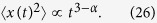


Note that the logarithmic correction is needed when *α* = 2. For case (3),


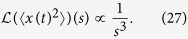


The inverse transform is


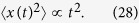


Therefore, superdiffusion is observed for cases (2) and (3), which is consistent with the Lévy walk.

When the persistence time is periodic, the MSD is given by


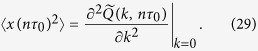


Because 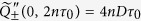 and 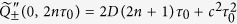, the MSD becomes


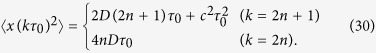


Because *D* is related to the bias, i.e. 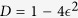, the bias decreases the diffusivity at *t* = 2*nτ*_0_. However, the MSD at *t* = (2*n* + 1)*τ*_0_ is greatly enhanced by a large period *τ*_0_ and bias 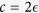.

We confirmed that the distribution of persistence time for the right jump follows a power-law distribution in the case of the chaotic time series (not shown). The power-law distribution means that there is no characteristic persistence time. This is one of the properties of superdiffusion shown in the Lévy walk^13^,^18^. However, the power-law distribution is not perfect in the case of the chaotic one (No. 180). The persistence-time distribution for the left jump does not follow a power law, whereas that for the right jump follows a power law in a short-time region. As a result, the overall property is classified as normal diffusion.

We can observe superdiffusion in our model if we find a time series of chaotic probability, such as the modified Bernoulli map (Aizawa map^19^,^20^), which generates power-law distributions of the persistence time. The Aizawa map is described by the following equations:


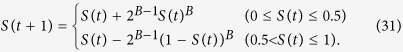


The persistence time on the interval [0, 0.5] or (0.5, 1] can be considered as a random variable because of the chaotic motions. It is known that the persistence-time distribution is given by a power-law distribution^20^,^21^:


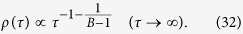


This distribution corresponds to the PDF *ρ(τ*) with 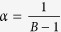 in the above theory. Using Eqs (24), (26) and (28), we obtain the time dependences of the MSD (=*t*^*β*^) for the Aizawa map, which is given by

- *B* ≤ 1.5: normal diffusion (*β* = 1.0),
- 1.5 < *B* ≤ 2.0: superdiffusion 
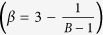
,
- *B* > 2.0: superdiffusion (*β* = 2.0).

**Figure 3:**
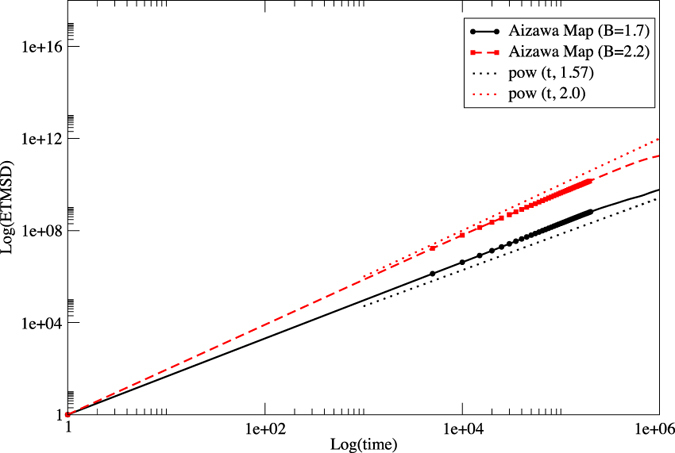
ETMSDs of a random walk using a time series generated by an Aizawa map with *B* = 1.7 (black circles) and *B* = 2.2 (red squares). The slope of each ETMSD indicates superdiffusion, which is very close to the expected value (1.57 or 2.0) from our theory.

Figure 3 shows the ETMSDs of a random walk using a time series generated by an Aizawa map with *B* = 1.7 and *B* = 2.2. Here, for the purposes of correctly estimating the slope, we do not use normalisation (Eq. (33)) and use 100 samples from different initial conditions. We successfully confirm that the exponent *β* indicates superdiffusion, which is very close to the expected value 1.57 or 2.0 from our theory.

## Discussion

To characterise the ‘chaotic probability’ observed in the nanoscale optical energy transfer between quantum dots (QDs)^6^, we investigated one-dimensional random walks with fluctuating biases generated by fixed, periodic, quasi-periodic, and chaotic time series. In this study, we demonstrated that the ETVAR clearly characterises the diffusive nature in random walks with fluctuating biases in the time-quenched framework, while the ETMSD does not estimate the diffusivity. Moreover, in the time-annealed framework, we showed that the MSD exhibits superdiffusion when the second moment of the persistence time diverges.

The definition of the ETVAR is similar to the detrended fluctuation analysis (DFA)^22^. In the DFA, the variance of the time series from which the linear fitting line (trend) is subtracted in each segment is analysed, whereas we used the time series from which the mean (bias) is subtracted, which is not linear in general, in each segment in the ETVAR. Therefore, the ETVAR can be used instead of the DFA to capture anomalous fluctuations in the time series when all time series are affected by the same trend (bias), as in the time-quenched framework.

This random walk with a temporally fluctuating bias is different from the previously studied Langevin equation with a fluctuating diffusivity, while the local (instantaneous) diffusivities in both models are fluctuating. The local diffusivity is generally determined by the surrounding environment and the shape of a diffusing particle. Therefore, the fluctuating diffusivity originates from the fluctuating shape of a particle or a fluctuating environment^23^, e.g. the centre-of-mass motion in the reptation model^24^ and diffusion in a heterogeneous environment^25^,^26^,^27^,^28^,^29^,^30^,^31^. On the other hand, the physical mechanism of the fluctuating bias is delayed feedback control in a photoexcitation transfer system or a change in the shape of the coin caused by collision deformation during coin tossing.

**Figure 4:**
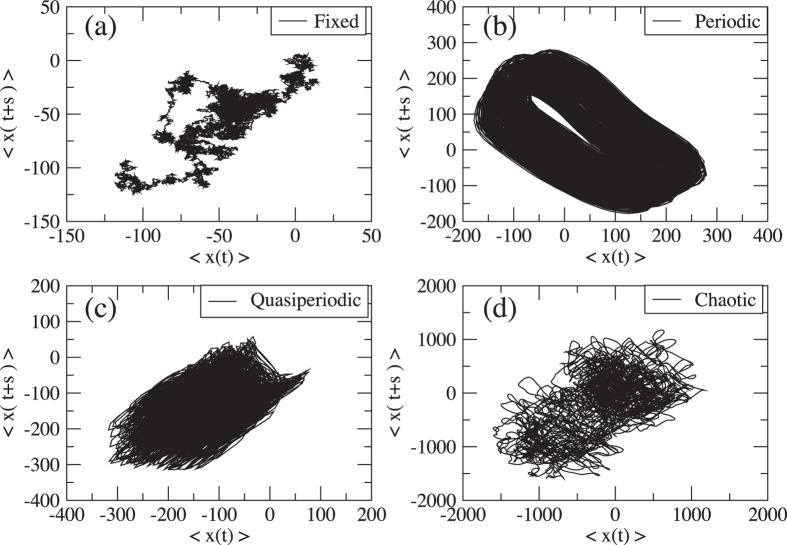
Average displacement 〈*x(t*)〉 versus 〈*x(t* + *s*)〉 for each time series.

Figure 4 shows the average displacement 〈*x(t*)〉 versus 〈*x(t* + *s*)〉 for each time series, where we chose *s* = 50,000. When the bias time series is chaotic, the figure becomes complex. It is noted that the large fluctuation in the mean displacement (MD) of No. 180 is only observed in the situation where the bias time series is quenched in time (see Fig. 1(b)). This large fluctuation allows for the possibility that a random walker arrives at some points (±*x*) in a short time. In real physical situations, nanophotonic oscillations are the oscillations of the existence probabilities of the optical energy in QDs but not a probability of the left flight of a particle. However, we believe that the properties of the chaotic probabilities could generate the short-time arrival as well. We expect that such a complexity and large fluctuation will provide new applications using nanoscale chaotic probabilities, such as efficient optical energy transfer used for information propagation, efficient decision-making devices, and high-quality and high-speed physical nanoscale RNGs for information and communications technologies.

## Methods

Here, we consider the random walk where the probability *p(t*) that the random walker jumps to the right at time *t* depends on *t*, i.e. a random walk with a fluctuating bias. The probability *p(t*) can be generated by dynamical systems, e.g. periodic, quasi-periodic, and chaotic time series or simply stochastic processes.

**Figure 5:**
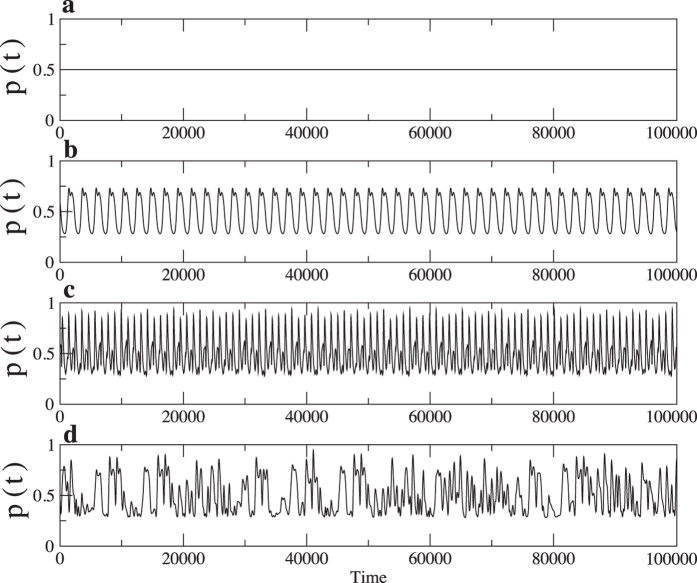
Normalised threshold time series: (**a**) fixed, (**b**) periodic (No. 30), (**c**) quasi-periodic (No. 207), and (**d**) chaotic (No. 180) time series. Here, the numbers denote the parameter numbers used in ref. 6.

To perform a Monte Carlo simulation of the random walk, we first prepare a good random number generator (RNG) which generates real values in [0, 1]. We used the Mersenne Twister (MT)^32^ in this study. When the value of the RNG at time *t* is smaller than *p(t*), *X(t*) = +1 (right flight); otherwise, *X(t*) = −1 (left flight). Thus, the trajectory *x(t*) = *X*(1) + 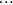 + *X(t*) represents the position of the random walker at time *t*. To construct *p(t*) with *p(t*) ∈ [0, 1] and 〈*p(t*)〉 = 0, we executed the following preprocessing for the time series *S(t*) (*t* = 1, 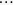, *T*):


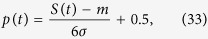


where *m* and *σ* are the mean and standard deviation of *S(t*), respectively. (*m* is 0.292 (periodic), 0.286 (qusi-periodic), and 0.239 (chaotic) for the original data.) Thus, the mean of *p(t*) is exactly 0.5, which implies that the net bias is zero. Figure 5 shows four different time series of *p(t*) obtained from a data set of nanophotonic oscillations^6^: e.g. (a) fixed, (b) periodic (No. 30), (c) quasi-periodic (No. 207), and (d) chaotic (No. 180) time series. In this study, we used these time series with *T* = 900,000, although only 100,000 time steps are shown in Fig. 5. Here, the numbers denote the parameter number used in ref. 6.

## Additional Information

**How to cite this article**: Kim, S.-J. *et al*. Random walk with chaotically driven bias. *Sci. Rep.* **6**, 38634; doi: 10.1038/srep38634 (2016).

**Publisher's note:** Springer Nature remains neutral with regard to jurisdictional claims in published maps and institutional affiliations.

## References

[b1] Bouchaud J. & Georges A. Anomalous diffusion in disordered media: Statistical mechanisms, models and physical applications. Phys. Rep. 195, 127–293 (1990).

[b2] Sinai Y. G. Limit behaviour of one-dimensional random walks in random environments. Theory Prob. Appl. 27, 247–258 (1982).

[b3] Patterson S. Dark Pools: The rise of the machine traders and the rigging of the U.S. stock market. (Crown Business; Reprint edition, 2013).

[b4] Weatherall J. O. The Physics of Wall Street: A Brief History of Predicting the Unpredictable (Mariner Books, 2013).

[b5] Malkiel B. G. A Random Walk Down Wall Street (W. W. Norton & Company Inc., rev. upd edition, 2016).

[b6] Naruse M., Kim S.-J., Aono M., Hori H. & Ohtsu M. Chaotic oscillation and random-number generation based on nanoscale optical-energy transfer. Sci. Rep. 4, 06039 (2014). 10.1038/srep06039 PMC4129418 25113239

[b7] Naruse M. . Spatiotemporal dynamics in optical energy transfer on the nanoscale and its application to constraint satisfaction problems. Phys. Rev. B 86, 125407 (2012).

[b8] Aono M. . Amoeba-inspired nanoarchitectonic computing: Solving intractable computational problems using nanoscale photoexcitation transfer dynamics. Langmuir 29, 7557–7564 (2013). 23565603 10.1021/la400301p

[b9] Kim S.-J., Naruse M., Aono M., Ohtsu M. & Hara M. Decision maker based on nanoscale photo-excitation transfer. Sci. Rep. 3, 02370 (2013). 10.1038/srep02370 PMC3738946 23928655

[b10] Naruse M. . Decision making based on optical excitation transfer via near-field interactions between quantum dots. J. Appl. Phys. 116, 154303 (2014).

[b11] Naruse M. . Single-photon decision maker. Sci. Rep. 5, 13253 (2015). 26278007 10.1038/srep13253 PMC4538607

[b12] Metzler R., Jeon J.-H., Cherstvya A. G. & Barkaid E. Anomalous diffusion models and their properties: non-stationarity, non-ergodicity, and ageing at the centenary of single particle tracking. Phys. Chem. Chem. Phys. 16, 24128–24164 (2014). 25297814 10.1039/c4cp03465a

[b13] Shlesinger M. F., Klafter J. & Wong Y. M. Random walks with infinite spatial and temporal moments. J. Stat. Phys. 27, 499–512 (1982).

[b14] Montroll E. W. & Weiss G. H. Random walks on lattices. II. J. Math. Phys. 6, 167 (1965).

[b15] Cox D. R. Renewal Theory (Methuen, London, 1962).

[b16] Akimoto T. Distributional Response to Biases in Deterministic Superdiffusion. Phys. Rev. Lett. 108, 164101 (2012). 22680721 10.1103/PhysRevLett.108.164101

[b17] Akimoto T. & Miyaguchi T. Phase diagram in stored-energy-driven Lévy flight. J. Stat. Phys. 157, 515–530 (2014). 10.1103/PhysRevE.87.062134 23848654

[b18] Geisel T., Nierwetberg J. & Zacherl A. Accelerated diffusion in Josephson junctions and related chaotic systems. Phys. Rev. Lett. 54, 616 (1985). 10031571 10.1103/PhysRevLett.54.616

[b19] Akimoto T. & Aizawa Y. Large fluctuations in the stationary and nonstationary chaos transition. Prog. Theor. Phys. 114, 737–748 (2005).

[b20] Aizawa Y. & Kohyama T. Symbolic dynamics approach to intermittent chaos - towards the comprehension of large scale self-similarity and asymptotic non-stationarity. In Chaos and Statistical Methods edited by Kuramoto Y. (Springer-Verlag, Berlin Heidelberg, 1983), pp. 109–116.

[b21] Aizawa Y., Murakami C. & Kohyama T. Statistical mechanics of intermittent chaos *f*^−*v*^ spectral behaviors of the semi-Markovian class. Prog. Theor. Phys. Suppl. 79, 96–124 (1984).

[b22] Peng C.-K. . Mosaic organization of DNA nucleotides. Phys. Rev. E 49, 1685–1689 (1994). 10.1103/physreve.49.1685 9961383

[b23] Rozenfeld R., Luczka J. & Talkner P. Brownian motion in a fluctuating medium. Phys. Lett. A 249, 409–414 (1998).

[b24] Ueyama T., Miyaguchi T. & Akimoto T. Fluctuation analysis of time-averaged mean-square displacement for the Langevin equation with time-dependent and fluctuating diffusivity, Phys. Rev. E 92, 032140 (2015). 10.1103/PhysRevE.92.032140 26465459

[b25] Manzo C. . Weak ergodicity breaking of receptor motion in living cells stemming from random diffusivity. Phys. Rev. X 5, 011021 (2015).

[b26] Massignan P. . Nonergodic subdiffusion from Brownian motion in an inhomogeneous medium. Phys. Rev. Lett. 112, 150603 (2014). 24785018 10.1103/PhysRevLett.112.150603

[b27] Yamamoto E., Kalli A. C., Akimoto T., Yasuoka K. & Sansom M. S. P. Anomalous dynamics of a lipid recognition protein on a membrane surface. Sci. Rep. 5, 18245 (2015). 26657413 10.1038/srep18245 PMC4677404

[b28] Akimoto T. & Seki K. Transition from distributional to ergodic behavior in an inhomogeneous diffusion process: Method revealing an unknown surface diffusivity. Phys. Rev. E 92, 022114 (2015). 10.1103/PhysRevE.92.022114 26382351

[b29] Chubynsky M. V. & Slater G. W. Diffusing diffusivity: A model for anomalous, yet Brownian, diffusion. Phys. Rev. Lett. 113, 098302 (2014). 25216011 10.1103/PhysRevLett.113.098302

[b30] Akimoto T. & Yamamoto E. Distributional behaviors of time-averaged observables in the Langevin equation with fluctuating diffusivity: Normal diffusion but anomalous fluctuations, Phys. Rev. E 93. 062109 (2016). 27415210 10.1103/PhysRevE.93.062109

[b31] Miyaguchi T., Akimoto T. & Yamamoto E. Langevin equation with fluctuating diffusivity: a two-state model. Phys. Rev. E 94, 012109 (2016). 27575079 10.1103/PhysRevE.94.012109

[b32] Matsumoto M. Mersenne Twister Home Page. http://www.math.sci.hiroshima-u.ac.jp/~m-mat/MT/emt.html.

